# Nebulization Therapy with Umbilical Cord Mesenchymal Stem Cell-Derived Exosomes for COVID-19 Pneumonia

**DOI:** 10.1007/s12015-022-10398-w

**Published:** 2022-06-04

**Authors:** Meiping Chu, Hao Wang, Linjie Bian, Jiehui Huang, Danping Wu, Ruiting Zhang, Fangli Fei, Yigang Chen, Jiazeng Xia

**Affiliations:** 1 Department of Respiratory and Critical Care Medicine, Wuxi Fifth People’s Hospital, Jiangsu, 214002 People’s Republic of China; 2 Cruilife Stem Cell Co. LTD, Fuan building, 727 Jiefang South Road, Wuxi City, Jiangsu 214002; Unit C-105, High-Tech Industrial Park, 70 Guxin Road, Chancheng District, Foshan, Guangdong 528010 People’s Republic of China; 3 Department of Radiology, The Affiliated Wuxi No. 2 People’s Hospital of Nanjing Medical University, Jiangsu, 214002 People’s Republic of China; 4 Department of General Surgery, The Affiliated Wuxi No. 2 People’s Hospital of Nanjing Medical University, 68 Zhongshan Road, Jiangsu, 214002 People’s Republic of China; 5 Wuxi Clinical College Affiliated to Nantong University, Wuxi, China

**Keywords:** COVID-19 pneumonia, Mesenchymal stem cells, Exosomes, Nebulization

## Abstract

**Background:**

Scientists have been facing numerous challenges in the development of an effective therapeutic strategy for the treatment of COVID-19 pneumonia. Several studies have suggested that improving patient immunity and reducing lung injury induced by SARS-CoV-2 may be effective for treating patients with COVID-19.

**Methods:**

A pilot trial of nebulization therapy with exosomes of mesenchymal stem cells (MSCs) was performed on seven patients with COVID-19 pneumonia. Exosomes secreted from MSCs were collected and purified using multiple ultrafiltration steps. All patients were treated with nebulization of MSC-derived exosomes, and primary safety and efficacy outcomes were evaluated.

**Results:**

Our clinical study demonstrated that nebulization of MSC-derived exosomes is a novel method that might be utilized in the treatment of COVID-19 pneumonia. Nebulization of MSC-derived exosomes did not induce acute allergic or secondary allergic reactions but did promote the absorption of pulmonary lesions and reduce the duration of hospitalization for mild cases of COVID-19 pneumonia.

**Conclusions:**

Nebulization of MSC-derived exosomes is a safe, effective, and simple method, and their application at the beginning of treatment may be more beneficial.

**Trial Registration:**

Chinese Clinical Trial Registry, ChiCTR2000030261. Registered on 26 February 2020.

**Graphical Abstract:**

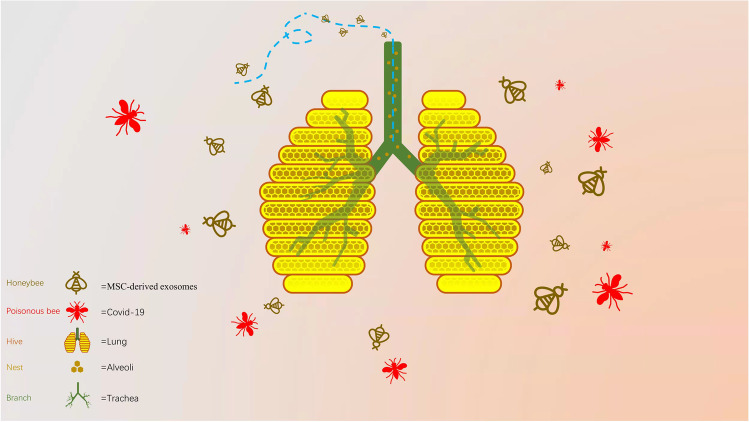

## Background

COVID-19 pneumonia has been declared a global pandemic by the World Health Organization (WHO) and has become a public health emergency [1]. Although the diagnostic efficiency and accuracy of treatment modalities have improved, the overall therapeutic effect remains limited [2]. The major causes of death include severe pneumonia, acute respiratory distress syndrome (ARDS), pulmonary oedema or multiple organ failure [3].

The two major characteristics of COVID-19 pneumonia are as follows: 1. those with low immunity are more likely to be infected with COVID-19 [4]; and 2. the major target organ is the lung [5]. Indeed, respiratory failure has been reported as one of the major causes of death due to COVID-19 [6], and autopsy has revealed pulmonary injury, significant exudative reaction, and pulmonary embolism in many of these patients [7]. Therefore, COVID-19-triggered lung injury might be reduced by improving the immunity of patients.

Mesenchymal stem cells (MSCs) have been shown to possess a comprehensive, powerful immunomodulatory function and regenerative function [8, 9]. MSCs can counteract cell death and promote cell regeneration associated with the pathogenesis of chronic obstructive pulmonary disease (COPD), idiopathic pulmonary fibrosis, asthma, ARDS, and pulmonary arterial hypertension [10, 11]. Moreover, MSC-secreted exosomes are shown to both regulate immunity through interacting with immune cells and inhibit the inflammatory response through cytokines [12, 13]. Numerous studies have demonstrated that MSC-secreted exosomes may be employed in the treatment of immune deficiency, inflammation, ARDS, and other lung diseases [14, 15]. Therefore, MSC-secreted exosomes may also be effective in treating COVID-19 pneumonia.

A routine method of stem cell therapy administration is intravenous injection [16, 17]. Exosomes are one of the major active components secreted by stem cells [18], with sizes ranging from 30–200 nm [19]. Exosomes can reach the bronchioles and alveoli directly after nebulization, which is conducive to maximal drug absorption [15]. Therefore, we hypothesized that nebulized MSC-secreted exosomes might be an effective treatment for COVID-19 pneumonia.

## Methods

### Study Design

A pilot trial of nebulization therapy for COVID-19 pneumonia with MSC-secreted exosomes of was performed on seven patients with COVID-19 pneumonia. The study was conducted at Wuxi Fifth People’s Hospital, China. The safety and scientific validity of this study have been issued in Chinese Clinical Trial Registry (ChiCTR2000030261).

### Inclusion Criteria

We initially enrolled patients with COVID-19 (age 18–65 years) according to the guidance of the National Health and Health Commission of China [20]. We comprehensively considered the patient's epidemiological history, clinical symptoms, and nucleic acid test results. Informed consent was obtained from all patients participating in this clinical study. Informed consents were obtained from all the patients who participated in this clinical study. The detailed inclusion criteria include: (1) Possess any single one of COVID-19 's epidemiological history and conforms to any two of the clinical manifestations (fever and/or respiratory symptoms; imaging features of the COVID-19 pneumonia; leukocyte count is normal or decreased and lymphocyte count is decreased in the early stage of the disease); (2) There was no clear epidemiological history of COVID-19 and conformed to 3 of the clinical manifestations (fever and/or respiratory symptoms; imaging features of the COVID-19 pneumonia; leukocyte count was normal or decreased and lymphocyte count decreased in the early stage of the disease); (3) Novel coronavirus (SARS-CoV-2) nucleic acid tested positive in respiratory tract or blood samples by real-time fluorescence PCR (RT-PCR); (4) Diagnosis and classification criteria of COVID-19 were in accordance with the Pneumonia Diagnosis and Treatment Program of Novel Coronavirus Infection (Trial Version, the 6th Edition,) published by the National Health Commission of the People’s Republic of China. According to the Program, the definitions of mild, moderate and severe forms of COVID-19 pneumonia are: (a) mild form refers to co-existing symptoms of mild clinical manifestation and no imaging; (b) moderate form refers to co-existing symptoms of fever, respiratory lesions and pneumonia revealed by X-ray or CT; and, (c) severe form refers to conditions that meet any of the followings: Respiratory distress, RR ≥ 30/min; Oxygen saturation ≤ 93% at rest state; Arterial partial pressure of oxygen (PaO_2_)/Fraction of inspiration O_2_ (FiO_2_) ≤ 300mnHg; (5) Between the ages of 18 and 65; (6) Understand the informed consent form, sign it, and volunteer to participate in this study.

### Exclusion Criteria

(1) Non-COVID-19 patients; (2) Ages < 18 or > 65; (3) Severe cardiovascular and cerebrovascular dysfunctions: severe cardiac rhythm abnormalities (such as ventricular arrhythmias and atrioventricular block requiring clinical intervention), unstable angina pectoris, myocardial infarction, severe valvular heart disease, refractory hypertension, NYHA cardiac function grade II or left ventricular ejection fraction (LVEF) < 50%, acute coronary syndrome, congestive heart failure, aortic dissection within 6 months, Stroke or other cardio-cerebrovascular episodes with grade 3 or above; (4) Severe hepatic and renal dysfunctions: total bilirubin (TBIL) > 3 ULN, alanine aminotransferase (ALT) or aspartate aminotransferase (AST) > 5 ULN, alkaline phosphatase (ALP) > 5 ULN and serum creatinine > 1.5 ULN; (5) pregnant and lactating women; (6) patients who were participating in other clinical trials or who have participated in other clinical trials in the last 3 months; (7) patients who were unwilling or unable to sign the informed consent due to illness.

### Patients

The patients were enrolled from February 26, 2020, to September 4,2020. All enrolled patients were confirmed to have COVID-19 via real-time reverse transcription polymerase chain reaction (RT-PCR) to detect SARS-CoV-2 RNA following the protocol outlined in a previous study [3, 21]. As stated under the Study design section, seven patients with COVID-19 pneumonia received exosome treatment. Since the COVID-19 outbreak was quickly contained in Wuxi where this study was located, no more patients were recruited to the treatment group. There were COVID-19 patients being admitted and subsequently discharged from the hospital. Among those patients, 39 of them were diagnosed of mild COVID-19 symptoms and were assign to a control group retrospectively.

**Fig. 1:**
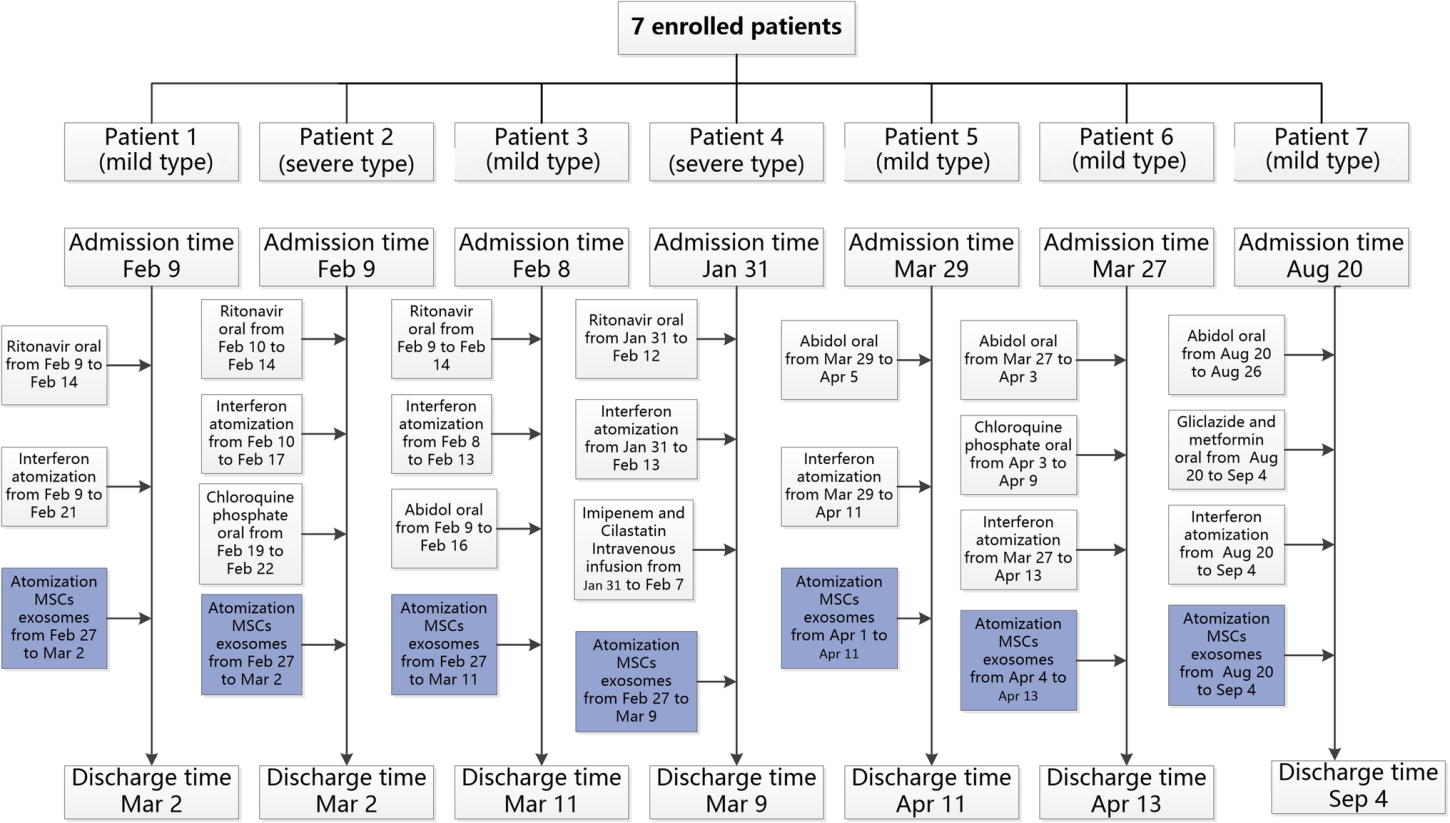
Flow chart showing the timeline of the nebulization treatment of MSC-derived exosome

All patients were treated with ritonavir orally, abidol orally, interferon nebulization, or chloroquine phosphate orally (Fig. 1). The clinical, laboratory, and radiological outcomes of all patients were recorded and certified by a trained group of doctors.

### Umbilical Cord Processing and Cell Isolation

Clinical grade MSCs were supplied, by Cruilife Stem Cell Co. LTD. Cruilife operates a stem cell banking and manufacturing facility that has been accredited by the American Association of Blood Banks (AABB). The donor screening, the procurement of the umbilical cord, the pathogen screening of the umbilical cord, the processing of the cord tissue, the culture of the MSC and the quality control overarching all these procedures are carefully managed and implemented. The Standard Operating Procedures for MSC isolation and culture are elaborated in the following [22].

An umbilical cord sample was collected aseptically, and was placed in a sterilized collection cup containing medium consisting of Dulbecco's Modified Eagle Medium (DMEM) with 4,500 mg/mL glucose and antibiotic solution containing 0.2% streptomycin, 0.1% gentamicin and 0.12% penicillin., The sample was put in a foam box precooled to 4 °C with blue ice and carried to the laboratory within 6 h of collection.

In a clean room, the umbilical cord sample in the cup was rinsed three times with PBS in order to remove blood clots. The sample was then placed in a Petri dish in a biosafety cabinet, which was cut into 5-cm-long segments using sterilized scissors and forceps. The segments were gashed longitudinally to expose the Wharton’s jelly and the blood vessels buried in; the Wharton’s Jelly was scraped away from the blood vessels and transferred in a separate Petri dish.

The Wharton’s jelly was cut into 1-to-2-mm-long pieces with scissors, and 5 ml of each piece was placed into a T175 culture flask. Then, 15 mL of complete medium (Mesenchymal Stem Cell Basal Medium, Cat# 6,114,011, Dakewe Bioengineering, China) with 5% cell culture supplement (EliteGro-Adv. GMP, Cat# EPAGMP-­500, EliteCell Biomedical, Beltsville, MD, USA) was added into the culture flask and was shaken gently so that the tissues distributed evenly. The sample was incubated at 37 °C in a 5% CO_2_ incubator for two days without disturbance such that the tissues adhered to the flask.

After five days of incubation, the culture medium was changed completely, and; cells outgrowing from the edge of the adherent explants were generally observed after approximately seven days of culture. Thereafter, the medium was changed every two to three days. When cell growth reached 80% confluence, which could be observed at six to seven days after the initial cell outgrowth from the explant, passaging of the cells was initiated. For passaging of cells, the flask was gently shaken so that the tissue pieces dissociated and were subsequently aspirated together with the supernatant.

The cells left in the flask were dissociated with four mL of commercially available trypsin solution and incubated at 37 °C for two minutes; five ml of complete medium was added to neutralize the solution. The cell suspension was then transferred to a centrifuge tube, and was centrifuged at 1,500 g for six minutes. The cell pellet was considered passage 0 (P0). For further cell expansion and characterization, P0 cells were suspended in complete medium, counted, and passaged at a seeding density of approximately 1.3 × 10^4^/cm^2^. Excess cells were cryopreserved as seed cells for further use.

### Characterization of Isolated Cells

Expression of cell surface markers was revealed analysed with flow cytometry: P5 cells displayed positive expressions of CD90, CD105, and CD73 and concurrent negative expressions of CD34, CD45, CD14, CD19 or HLA-DR. Meanwhile, P5 cells displayed chondrogenic differentiation, adipogenic differentiation and osteogenic differentiation potentials when cultured in appropriate differentiation media.

All procedures performed in this study involving human samples were in accordance with the ethical standards of the institutional research committee and the guidelines set by the Declaration of Helsinki.

### Isolation and Characterization of Exosomes Isolated from MSCs

**Fig. 2:**
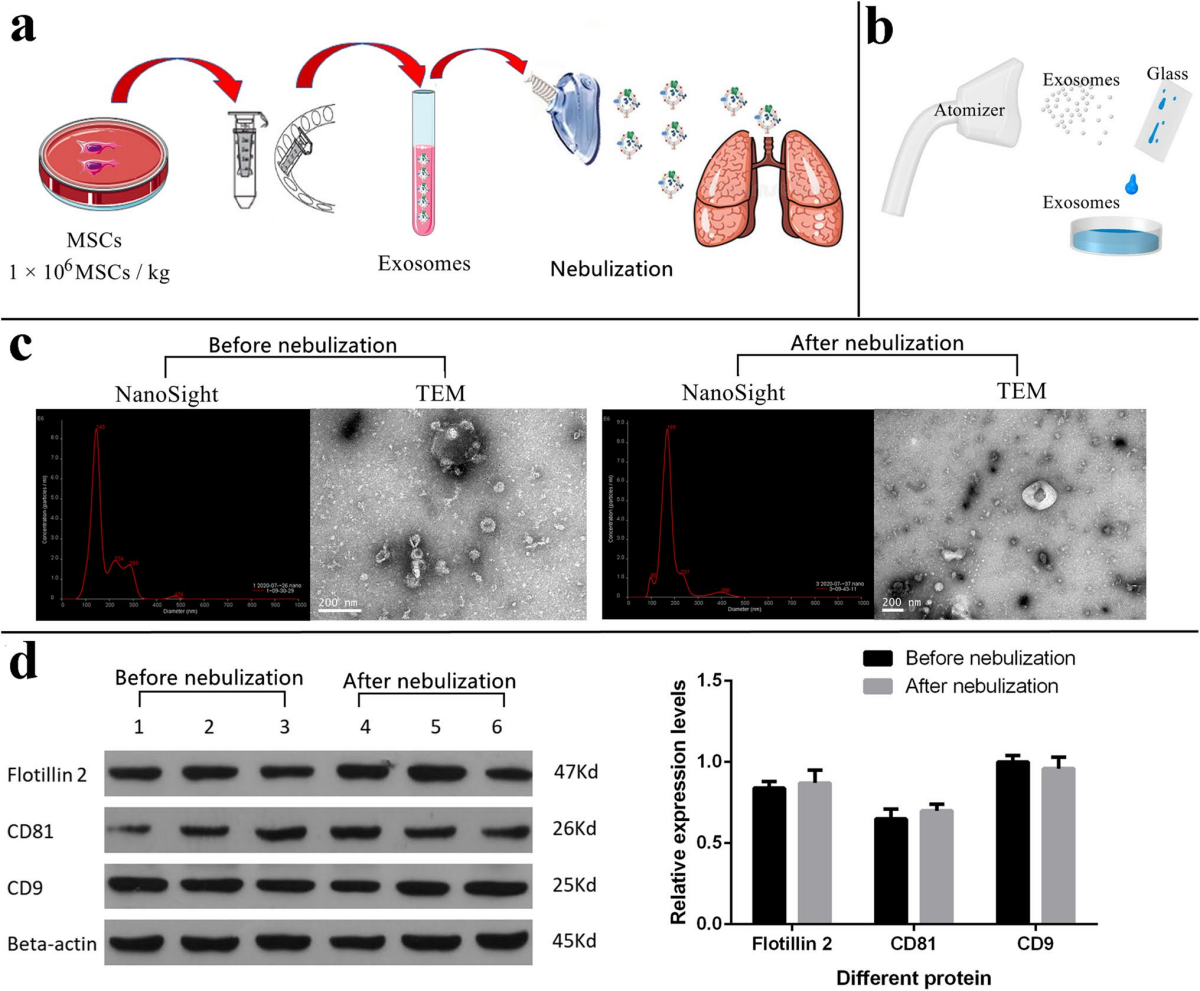
Extraction and characterization of MSC-secreted exosomes. (**a**) Schematic diagram of nebulization treatment of MSC-derived exosome exosomes. (**b**) Schematic diagram of the exosome collecting device after nebulization. (**c**) Exosome samples were analyzed by nanoparticle tracking analysis and transmission electron microscopy. (**d**) Exosome samples were confirmed by immunoblotting for known exosome markers CD9, CD81 and Flotillin 2

MSC-secreted exosomes s were collected and purified using multiple ultrafiltration steps (Fig. 2a). The secretomes were first centrifuged at 4 ℃ at 3,000 × g for 20 min and filtered through a 0.22 μm filter to remove any cells or cell debris. The filtered secretomes were then placed in a new sterile EP tube, followed by the addition of 0.2 ml exosome separation and purification solution (Shanghai Gefan Biotechnology Co., Ltd. Product No.: ex010). The contents were kept at 4 ℃ overnight for sufficient mixing and then centrifuged at 4 ℃ at 3,000 × g for 20 min on the following day. After the supernatant was aspirated, it was centrifuged at 4 ℃ for 1,500 × g for 5 min to remove the residual solution. After 1,000 μl sterile PBS being added to resuspend the exosomes, the suspension was centrifuged for 1 h at 100,000 × g using an ultracentrifuge, and this procedure was repeated three times. The exosome samples were analyzed analysed for proper size using nanoparticle tracking analysis (NTA; NanoSight NS300, Malvern) and for morphology using transmission electron microscopy (TEM; Tecnai G2 Spirit Bio TWIN). For the confirmation of successful exosome isolation, standard Western blotting was performed for known exosome markers were performed using rabbit antibodies against human CD81 (1:6000, ab109201, Abcam), CD9 (1:2000, ab92726, Abcam), and Flotillin 2 (1:6000, ab181988, Abcam). Protein sample loading was monitored by probing the same membrane filter with an anti-β-actin antibody (1:2000, sc-130301, Santa Cruz) (Fig. 2).

The nebulized MSC exosomes were sprayed onto a sterile glass plate, and a dish was placed under the glass plate for collecting the liquid containing the exosomes. After nebulization, the size and markers of the MSC exosomes were evaluated again. No significant difference in the size and markers of MSC exosomes prior to and after nebulization was observed (Fig. 2c and Fig. 2d).

### Nebulization Treatment of MSC-Derived Exosomes

After obtaining ethical approval, all patients diagnosed with COVID-19 pneumonia were provided informed consent and were treated with nebulized MSC-derived exosomes. In a phase I clinical trial in which intravenous infusions of MSCs were used to treat ARDS, J.G. Wilson et al. started with a low dose of MSCs (1 million cells/kg predicted body weight) [23]. As a reasonable starting point, the doses of the exosome were chosen to be those secreted by the same amount of MSCs proportional to the patients’ body weight (1 million cells/kg predicted body weight). The concentration of exosomes for nebulization for each patient ranged from 7.66e + 0.8 to 7.00e + 0.7 particles/ml based on NanoSight. The exosomes derived from MSCs were diluted to 5 ml with 0.9% sodium chloride and added to an atomizer (Emedical, Excellentcare Medical Ltd. China). Nebulization treatments were performed twice a day (am 8:30, pm 16:00) for 10 min each. The patients were assessed by investigators after receiving the nebulization treatment.

### Treatment Procedure of MSC-Derived Exosomes and General Patient Information

This study was conducted from February 26, 2020 to September 4, 2020. The seven patients diagnosed with COVID-19 pneumonia, including two severe cases (patients 2 and 4) and five mild cases (patients 1, 3, 5, 6 and 7), were enrolled in the study (Table 1). Patients 1, 2, 3 and 4 underwent nebulization treatment of MSC-derived exosomes after antiviral treatment for a period of time. Patient 1 was a mild case and did not have any underlying disease conditions. Patient 2 was a severe case and had a comorbidity of liver function damage. Patient 3 was a mild case and patient 4 a severe case, and both did not have any underlying disease. Patients 5, 6 and 7 underwent nebulization treatment of MSC-derived exosomes from the beginning of treatment. Information about all the treatment modalities of the patients was collected. The timepoints of the nebulization treatment of MSC-derived exosome for each patient are shown in Fig. 1.

**Table 1:** General information of the enrolled patients

	Patient 1	Patient 2	Patient 3	Patient 4	Patient 5	Patient 6	Patient 7
Sex	F	M	F	F	M	M	M
Age (years)	62	53	23	61	43	19	57
Underlying diseases	No	Liver damage	No	No	Fatty liver	No	Diabetes mellitus
COVID-19 type	Common	Severe	Common	Severe	Common	Common	Common
Fever (°C, baseline)	38.5	37.4	37.6	38.9	37.7	37.4	37.6
Cough	Yes	Yes	No	Yes	No	No	Yes
Weakness	Yes	No	No	Yes	No	No	No
Diarrhea	No	Yes	No	No	No	No	No
Shortness of breath	No	No	No	Yes	No	No	No
Chest tightness	Yes	Yes	No	No	No	No	No
Oxygen saturation at rest state (%)	87	74	93	61	97	97	98
Date of diagnosed	Feb 9	Feb 9	Feb 8	Jan 31	Mar 28	Mar 27	Aug 20
Date of exosome treatment	Feb 27	Feb 27	Feb 27	Feb 27	Apr 1	Apr 4	Aug 23
First testing time for CRP after exosome treatment	Feb 28	Feb 28	Mar 3	Mar 9	Apr 4	Apr 8	Aug 25
Date of recovery	Mar 2	Mar 2	Mar 11	Mar 9	Apr 11	Apr 13	Sep 4
Hospital day	22	22	31	38	14	17	15

Yes: Presence of characteristic clinical symptoms, such as cough, weakness, diarrhea, shortness of breath and chest tightness

No: No characteristic clinical symptoms

CRP: Plasma C-reactive Protein

### Primary Outcomes

The primary outcomes of this study were the safety data consisting of secondary infection, allergic reactions, and life-threatening adverse events.

### Secondary Outcomes

The secondary efficacy outcomes consisted of the total white blood cell count, total lymphocyte count (tested by a Hitachi 7600–020 automatic biochemical analyzer), SARS-CoV-2 nucleic acid testing (tested by a RT-PCR protocol, DAAN GENE Co., Ltd, China), chest computerized tomography (tested by a 320-slice spiral CT scanner, Aquilion One, Toshiba Medical System, Japan), respiratory rate, adverse effects (fever, shortness of breath, and etc.), and duration of hospitalization. Because of the limitations in the study design, though some sort of efficacy has been observed for COVID-19 pneumonia, the oxygen saturation and level of CRP in plasma, for example, statistical significancy of the data is low; only a double-blind, randomized controlled trial in the future can yield a definitive answer.

### Statistical Analysis

Statistical analyses were analyzed using SPSS software (SPSS 22.0). Differences between two groups were assessed using chi square tests or unpaired two-tailed *t* tests based on the type of data. Data involving more than two groups were assessed using analysis of variance (ANOVA). A P-value of 0.05 or less was considered statistically significant.

## Results

### The Primary Safety Outcome

No acute allergic reactions such as swelling of the throat or tongue, itchy rash, shortness of breath, lightheadedness, vomiting, or low blood pressure, were observed within two hours after the nebulization treatment. Secondary allergic reactions were not observed posttreatment either. No adverse events were reported.

MSC therapy via intravenous infusion is considered safe for pulmonary diseases, such as COPD, ARDS, and idiopathic pulmonary fibrosis (IPF) [24, 25]. Several completed phase I trials have reported no acute, serious, or adverse events for MSC therapy [23, 26]. Several pioneering studies have been conducted on the safety of MSC exosome therapy. The findings of these studies suggested that MSC-derived exosomes were safe in the treatment of pulmonary diseases [27]. However, the routes of administration of MSC-derived exosomes in most studies were through intravenous infusion. Our results demonstrated that nebulization treatment of MSC-derived exosomes is safe and can be employed in the treatment of pulmonary diseases.

### The Efficacy Outcome

The CRP level of patient 1 decreased from 88.4 mg/L (Feb 17) to 4.3 mg/L (Feb 28) and 0.4 mg/L (Mar 1, 2020). The CRP level of patient 2 (severe case) decreased from 30.8 mg/L (Feb 23) to 18.9 mg/L (Feb 28, 2020) and 3.5 mg/L (Mar 2, 2020); for patient 3, it decreased from 5.7 mg/L (Feb 15) to 0.5 mg/L (Mar 3, 2020). The CRP level of patient 4 (severe case) decreased from 31.8 mg/L (Feb 25) to 11.4 mg/L (Mar 9, 2020). The nebulization treatment for patients 1, 2, 3, and 4 initiated on February 27, 2020. For patient 5, the CRP level was 0.5 mg/L on Mar 28 and remained the same onApril 4, 2020. However, the CRP level decreased from 2.2 mg/L (March 30) to 0.5 mg/L (April 8, 2020) for patient 6. The nebulization treatment for patients 5 and 6 initiated on April 1, 2020 and April 4, 2020, respectively. For patient 7, the CRP level were 1.9 mg/L (August 19), 5.4 mg/L (August 25) and 3 mg/L (August 29, 2020) (Table 2). The nebulization treatment for patients 5 and 7 initiated on April 1 and August 20. Although the CRP levels prior to the nebulization treatment (23.00 ± 31.87) was higher than those after treatment (3.17 ± 4.12), the difference was not statistically significant (*p* = 0.151).

**Table 2:** Efficacy outcomes before and after nebulization treatment

Laboratory index	Patient 1 (common type)		Patient 2 (severe type)		Patient 3 (common type)		Patient 4 (severe type)		Patient 5 (common type)		Patient 6 (common type)		Patient 7 (common type)		*P* value
	Pre-	Post-	Pre-	Post-	Pre-	Post-	Pre-	Post-	Pre-	Post-	Pre-	Post-	Pre-	Post-	
C-reactive protein (mg/L)	88.4	0.4	30.8	3.5	5.7	0.5	31.8	11.4	0.5	0.5	2.2	0.5	1.9	5.4	0.151
White cell count (*10^9^ per litre)	5.28	4.96	5.99	5.02	7.28	6.87	6.38	3.95	8.16	5.26	6.02	8.41	4.34	5.63	0.479
Lymphoma count (*10^9^ per litre)	3.6	2.91	1.36	2.03	2.87	2.62	1.17	1.1	2.77	2.36	2.08	2.08	3.08	2.95	0.770
Respiratory rate (/min)	12	13	32	25	15	14	22	20	14	15	16	15	13	14	0.723
Fever (°C)	36.5	36.6	36.7	36.5	36.3	36.4	37.1	37	36.5	36.6	36.4	36.5	37.6	36.5	0.468
Shortness of breath	No	No	Yes	No	No	No	No	No	No	No	No	No	No	No	

Pre-: Before nebulization treatment

Post-: After nebulization treatment

No: No shortness of breath

Yes: Shortness of breath

The oxygen saturation level without supplementation rose from 95% (Feb 26) to 98% (Mar 2) for patient 1 increased from 95% (Feb 26) to 98% (Mar 3) for patient 2 (severe case), and rose from 95% (Feb 26) to 100% (Mar 1) for patient 4 (severe case); whereas the changes in the oxygen saturation levels for patients 3, 5, 6 and 7 were negligible after the nebulization treatment. Therefore, no significant differences in the oxygen saturation level were found before and after nebulization treatment of MSC exosomes (*p* > 0.05).

Additionally, no significant differences were found in the total white blood cell count, total lymphocyte count, fever or shortness of breath before and after the nebulization treatment (*p* > 0.05). The alanine aminotransferase (ALT) of patient 2, which was a severe case with a comorbidity of liver function damage, decreased from 168 µ/l (Feb 26) to 92 µ/l (Feb 28) and 52 µ/l (Mar 2, 2020).

**Fig. 3:**
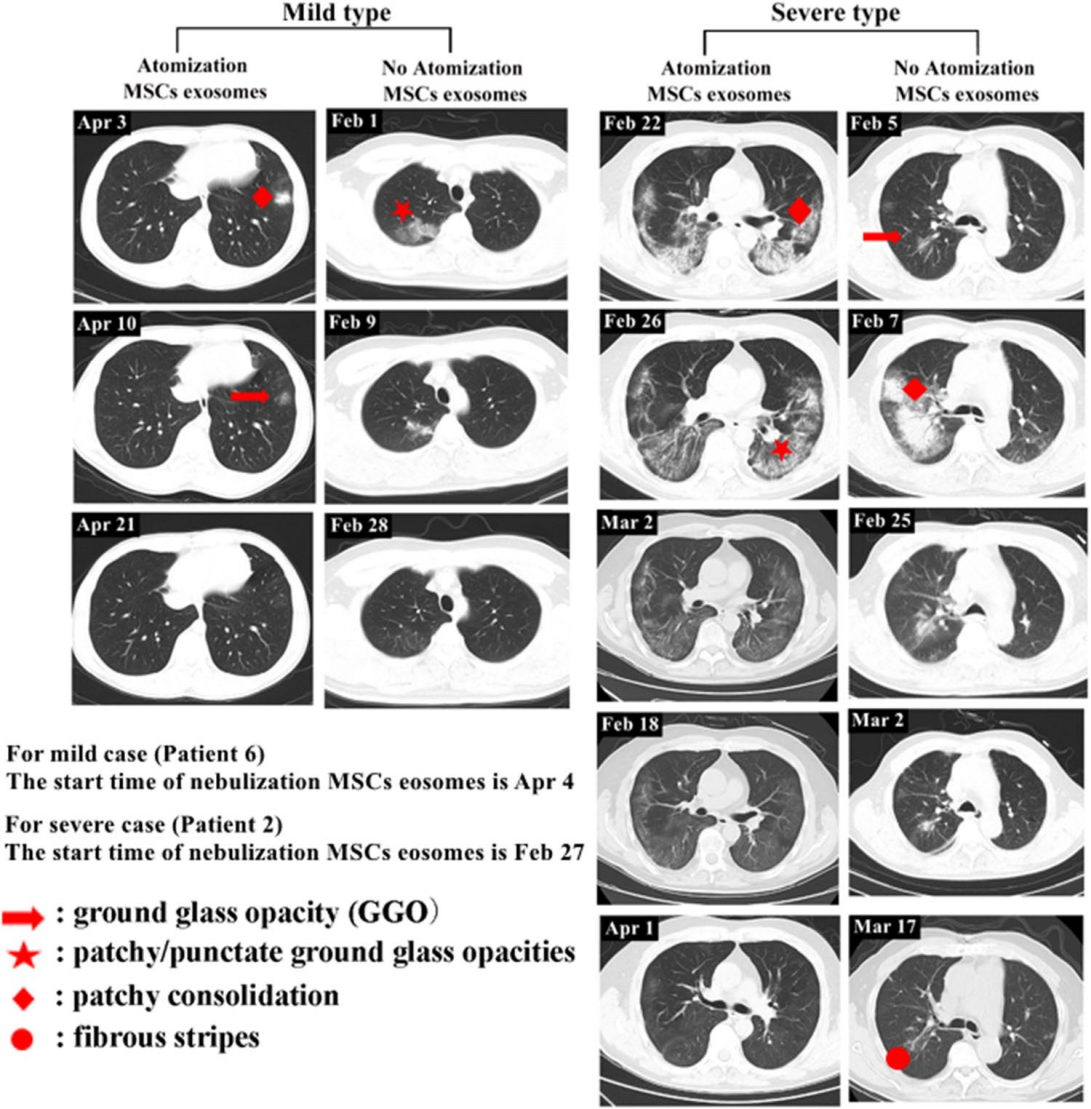
Chest CT images of mild and severe cases of COVID-19 pneumonia. (**a**) On April 3, 2020, the first CT scan of patient 6 showed an isolated nodule outside the left lower lobe of the lung. On April 4, the patient received nebulization treatment. On April 10, the second CT examination showed that the density of the left inferior lobe nodule reduced significantly, and its size also shrank. On April 21, the third CT examination showed that the lesions in the lower left lung were completely absorbed. The absorption time of similar lung lesions in another patient who received no exosome treatment was slower than patient 6. (**b**) Patient 2 received exosome treatments from February 27, 2020, and on March 18, the CT scan of patient 2 revealed obvious absorption of lesions in both lungs as well as fading density of the lesions. On April 1, the pulmonary lesions of this patient were completely absorbed. For the patients who did not receive exosome treatment, the presence of a fibrous cord shadow remained after absorption of the pulmonary focus

**Fig. 4:**
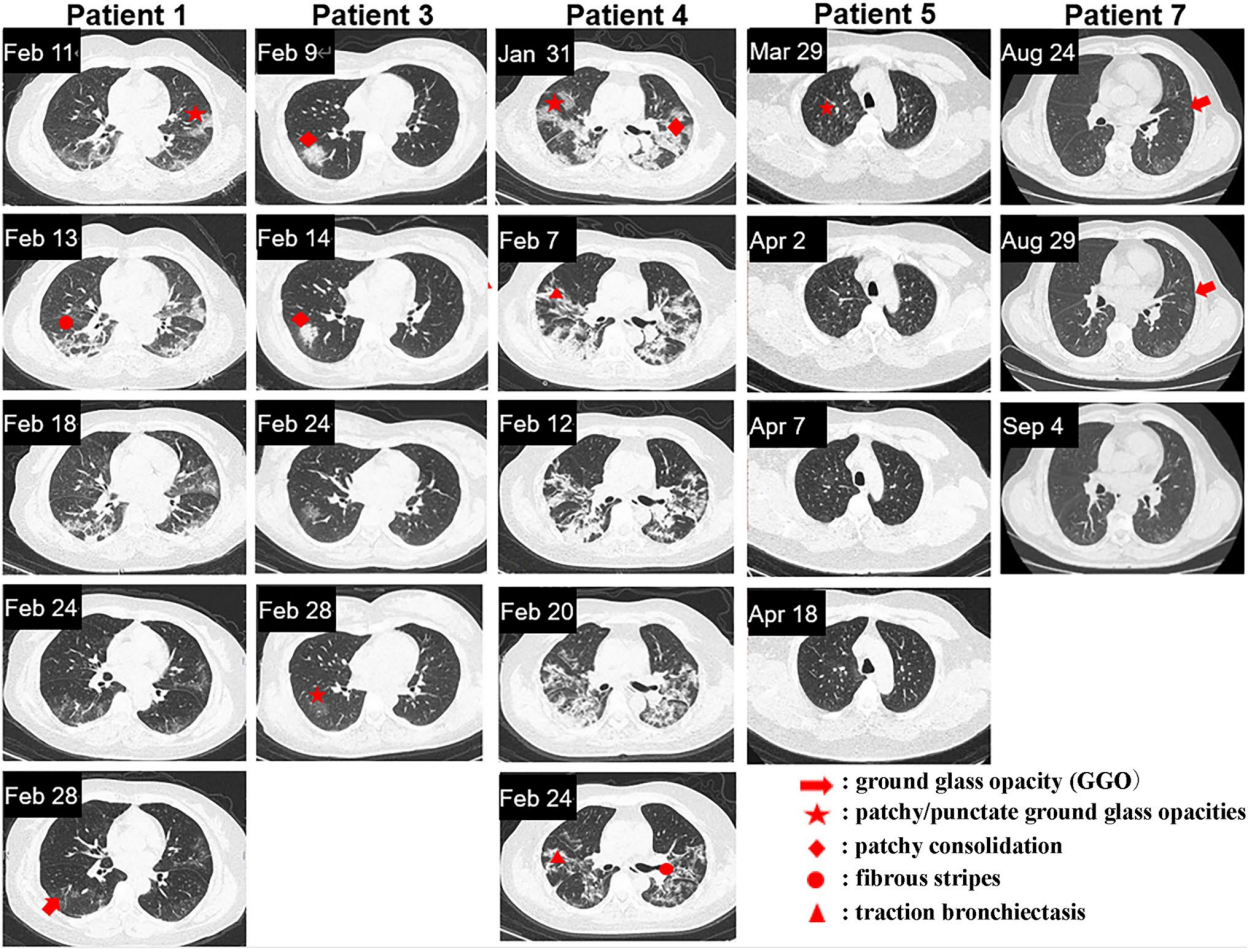
Chest CT images of patients 1, 3, 4, 5 and 7. The exosome treatments for patients 1, 3, and 4 started on February 27, 2020. The exosome treatments for patients 5 and 7 started on April 1, 2020 and August 20, 2020, respectively

The chest CT examinations indicated that the nebulization treatment of MSC-derived exosomes promoted the absorption of pulmonary lesions. On April 3, the first CT examination of patient 6 (mild case), revealed an isolated nodule outside the left lower lobe of the lung. On April 10, the second CT examination revealed that the density of the left inferior lobe nodule significantly redued, and its size shrank too. On April 21, the third CT examination revealed that the lesions in the lower left lung were completely absorbed. The time required for complete absorption of pulmonary lesions for patient 6 was 18 days. Comparatively, the absorption time of similar lung lesions in another patient (mild case, no nebulization treatment) was 27 days. Indeed, for patients with mild COVID-19 pneumonia, a significant difference was discovered in the time of complete absorption of pulmonary lesions (16.00 ± 5.23 *vs* 20.85 ± 3.57 days, *p* = 0.033) between those who received the exosome treatments from the beginning and those who received no exosome treatments, or, only received the exosome treatment once at the last stage before all modalities of treatments were terminated. The second group of patients consists of 39 cases of mild COVID-19 patients. Among them, 36 never received exosome treatment and the rest received exosome treatment once one day before they were discharged from the hospital. Patients with severe cases of COVID-19 pneumonia received exosome treatments at the later stage of treatment. Compared to those who received no exosome treatments, patient 2 showed obvious absorption of pulmonary lesions. In addition, fibrous shadows were present in the lung lesions of patients who received no exosome treatments (Fig. 3). CT images of the other five patients are shown in Fig. 4.

Several pioneering studies have demonstrated that bone marrow-derived exosomes can mitigate lung inflammation, and can alleviate pulmonary oedema as well as post-inflammatory complications in animal models of acute lung injury, asthma, ARDS and other inflammatory diseases [28–30]. MSC-derived exosomes contain bioactive substances such as mRNAs, miRNAs and proteins [31, 32], and these substances have been shown to effectively reduce inflammations and modulate airway remodeling [15, 33]. We also demonstrated that MSC-derived exosomes are capable of reducing the CRP level in patients with different degrees of COVID-19 pneumonia, in consistence with the findings of a previous study [20].

Despite the observed reduction in CRP level after the exosome treatment, statistical significance was not met due to the small number of enrolled patients and large standard deviation. Nevertheless, the patients who received exosome treatment at an earlier stage gained more benefits in terms of pulmonary lesion absorption. Our results indicated that nebulization treatment of MSC-derived exosomes improved the absorption of pulmonary lesions in mild cases of COVID-19 pneumonia and reduced cellular residue in severe cases.

### Duration of Hospitalization

The average duration of hospitalization was 18.74 ± 4.72 days for all COVID-19 patients, 18.29 ± 4.60 days for patients of mild cases, and 22.6 ± 4.3 days for patients of severe cases. The mean duration of hospitalization for patients 5, 6 and 7(mild cases who received nebulization treatment of exosomes from the beginning) was 15.3 ± 1.33 days (14, 17 and 15 days, respectively). The duration of hospitalization for patients 1 and 3, with mild cases of COVID-19 (who received nebulization of exosomes at the end of treatment), was 22 and 31 days, respectively. For the patients of mild cases, there was a significant difference in the duration of hospitalization (p = 0.035) between the group who received exosome treatment from the beginning and those who received no exosome treatment or only received exosome treatment once at very late stage. The duration of hospitalization for patients 2 and 4 (severe cases, exosome treatments at the last stage) were 22 and 38 days, respectively. Patients of mild cases who received exosome treatments at an earlier stage gained more benefits in terms of the duration of hospitalization. It should be emphasized that these results are for mild cases. None of the severe cases received exosome treatments in the early stage after they were hospitalized; therefore, it is unclear whether the timing of the exosome treatments affected their duration of hospitalization.

### COVID-19 Nucleic Acid Tests

Real time-PCR was performed using the genomic materials of patients with COVID-19 pneumonia. For all the enrolled patients, negative results of nucleic acid tests were required before exosome treatments started.

### Serum Immune Factor Analysis

Patient 6 was tested for immune factors including IL-2, IL-4, IL-6, IL-10, TNFα, IFN-γ, IL-17A, CD3, CD4, CD8, CD4/CD8, TH19 and NK, and the largest variation before and after exosome treatments was observed for IFN-γ. A twofold increase in IFN-γ, IL-17A and TH19 were observed after exosome treatments. Meanwhile, a twofold decrease was observed for the NK cells after exosome treatments (Table 3).

**Table 3:** Analysis of serum immune factors in patient 6. Nebulization was performed on April 4

Date	IL-2 (pg/ml)	IL-4 (pg/ml)	IL-6 (pg/ml)	IL-10 (pg/ml)	TNF-α (pg/ml)	IFN-γ (pg/ml)	IL-17A (pg/ml)	CD3 (%)	CD4 (%)	CD8 (%)	CD4/CD8	TH19 (%)	NK (%)
Before (March 30)	2.19	2.05	3.25	2.76	1.94	0.63	0	55.5	25.88	23.13	1.12	7.56	31.04
After (April 8)	No	No	3.54	0.83	No	No	No	67.62	33.52	27.64	1.21	14.40	14.68
After (April 13)	0.51	3.65	4.57	2.13	1.01	41.23	4.73	64.91	33.98	25.72	1.35	15.15	15.20

Before: Before exosome treatment

After: After exosome treatment

No: No data

IFN-γ is a common, important target of immunotherapy. Studies have shown that IFN-γ correlated to the disease severity, treatment efficacy and prognosis of COVID-19 pneumonia [34, 35]. Utilizing the potential therapeutic effect of IFN-γ to manage coronavirus-induced cytokine release syndrome should be considered for clinical application [36]. In this study, levels of IFN-γ increased obviously after exosome treatment. However, more studied are needed to confirm this mechanism of action.

Although several modalities have been proposed for the treatment of COVID-19 pneumonia, only a few of them are effective. At present, it is believed that reducing the lung injury at early stage of infection may be the key to saving these patients. Our results suggest that nebulization treatment of MSC-derived exosomes can promote the absorption of pulmonary lesions and reduce the duration of hospitalization for mild cases of COVID-19 pneumonia. On top of that, nebulization treatment of MSC-derived exosomes at early stage of infection may be more beneficial to patients.

Previous studies reported that exosomes secreted by MSC exert their therapeutic effects via several distinct mechanisms. These include a number of functional RNAs and proteins. Exosomes have an inherent targeting ability to deliver cargo RNAs to other cells [37]. These RNAs include IL-10 mRNA [38], miR-126 [39, 40] and miR-30b [41], which have been reported to protect lung fibrosis [42–45]. The other category of bioactive payload of exosomes are various immunomodulatory proteins. These proteins include angiopoietin-1 (Ang-1) [46] and hepatocyte growth factor (HGF) [47]. Both proteins are known to attenuate lung injury in animal models [48, 49]. Two useful online databases that assemble a large body of EV/exosome data are Exocarta [50] and Vesiclepedia [51].

In general, drug nebulization is an effective way to treat lung diseases. Nebulized exosomes can reach the fine structure of the lung, like the bronchiole and the alveoli, to directly deliver the anti-inflammatory molecules to their targets. Another reason that the nebulization approach was chosen over intravenous administration was because the latter, though may benefit multiple organs in addition to the lung, would introduce some interference factors into the current study and therefore renders the study less manageable. To date, there are only a few reports on the nebulization treatment of MSC-derived exosomes. Our results indicate that this treatment modality is a safe and feasible therapeutic approach for the treatment of patients with COVID-19 pneumonia. After the extraction of exosomes, they can be stored up to a week at 4 °C and may be added to an existing nebulizer when needed. The relatively long shelf life and easier administration approach of the exosomes, in comparison with MSCs, are crucial to COVID-19 pneumonia therapy. This is especially important in countries and regions lacking advanced health-care facilities.

However, our study has obvious shortcomings. Because of the short duration of the Delta-variant triggered COVID-19 outbreak in China in 2020, only seven COVID-19 pneumonia patients were included in this study. During the rest of 2020, no more COVID-19 patients were admitted to the hospital where this clinical study were carried out (Wuxi Fifth People's Hospital is the only hospital in Wuxi which is authorized to admit COVID-19 patients). In addtion, only three patients received the nebulization treatment of MSC-derived exosomes from the beginning of treatment. It should also be noted that we have not compared the nebulization method with other administration methods in this study, such as intravenous injection.

## Conclusions

In conclusion, our clinical study provides preliminary safety data of the MSC exosome treatment through nebulization as a novel approach of delivering exosomes to patients. In addition to the safety outcomes, some sort of efficacy is observed for patients of mild cases of COVID-19 pneumonia; however, the efficacy outcome is not robust owning to the lack of randomization and double blindness in the study design. Based on the lessons we learned from the current study, we are ready to conduct a larger-scale, better-designed clinical trial should another COVID-19 epidemic occurs in the future.

## Data Availability

The datasets generated during and/or analysed during the current study are available from the corresponding author on reasonable request.

## Abbreviations

MSCs: Mesenchymal stem cells
WHO: World health organization
ARDS: Acute respiratory distress syndrome
COPD: Obstructive pulmonary disease
